# Supplementary material for: Longitudinal Study on the Effect of Season and Weather on the Behaviour of Domestic Cats (Felis catus)
Source: Animals (Basel). 2025 Feb 22;15(5):637. doi: 10.3390/ani15050637 (PMC11898201; doi:10.3390/ani15050637)
Supplement: Supplementary file 1 [file animals-15-00637-s001.zip › animals-3465554-supplementary.pdf]

Supplementary Material

Supplementary Material 1 – Boxplots of individual cats

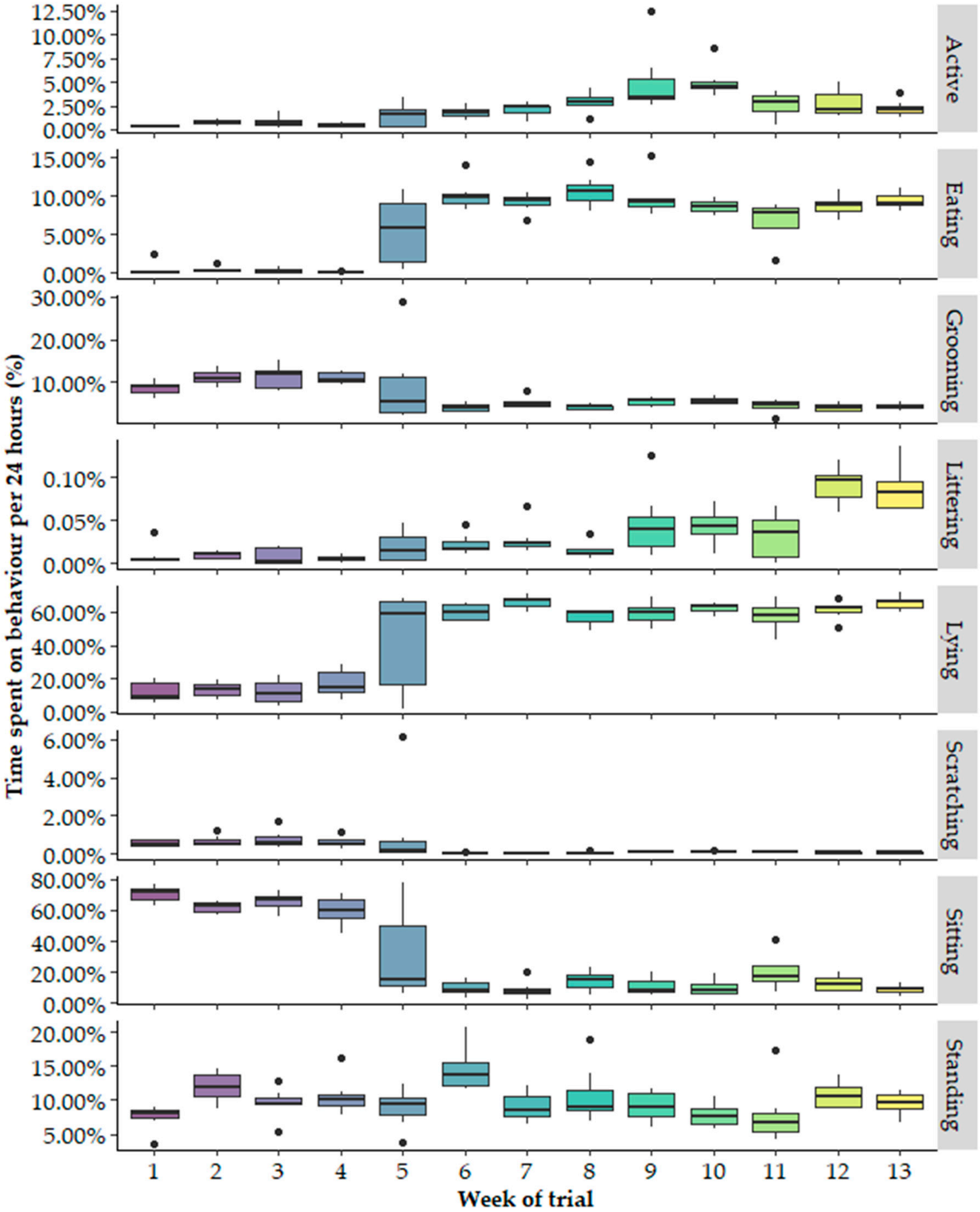

Figure S1. Boxplots of daily proportional behaviour data for Cho.

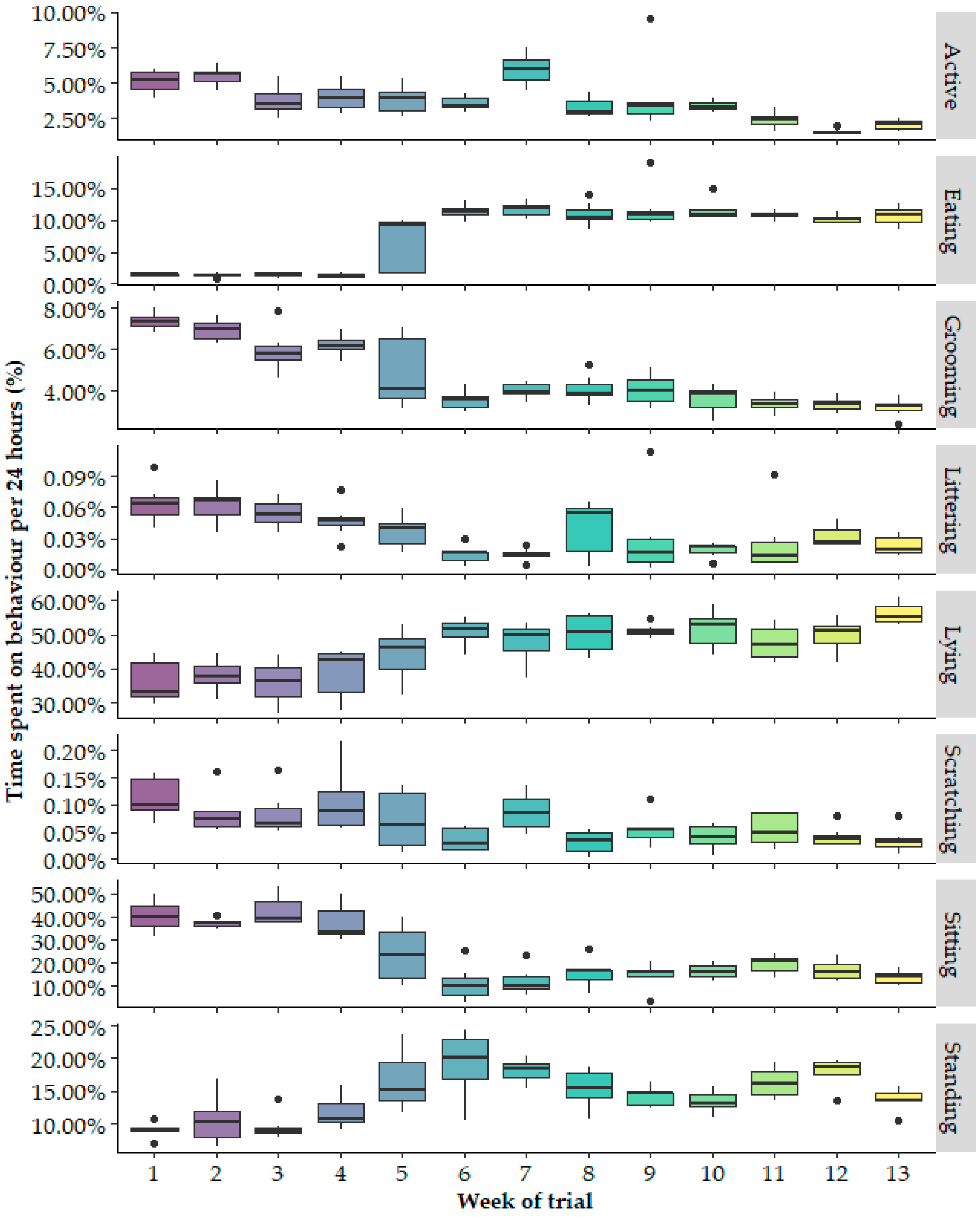

Figure S2. Boxplots of daily proportional behaviour data for George.

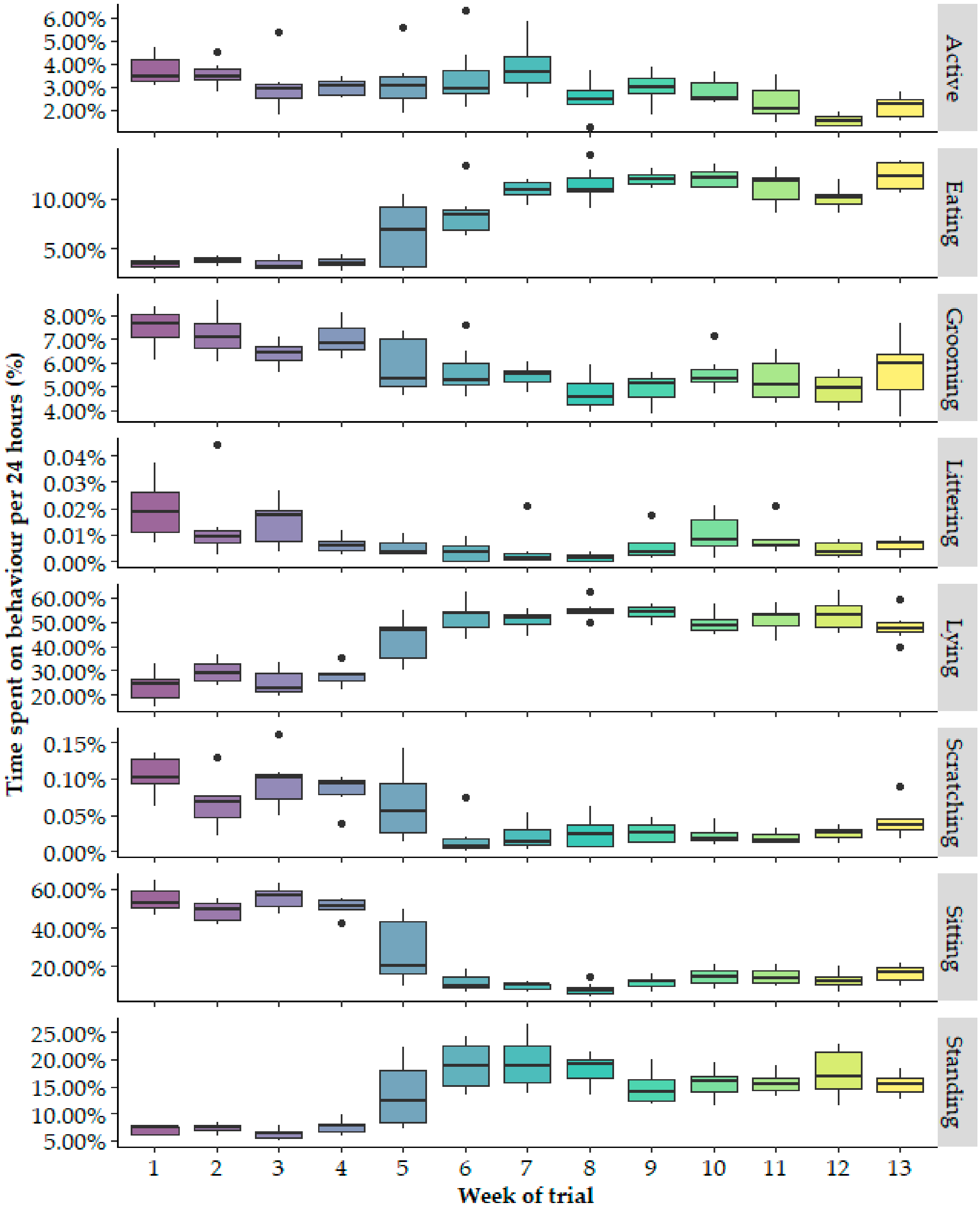

Figure S3. Boxplots of daily proportional behaviour data for Hagrid.

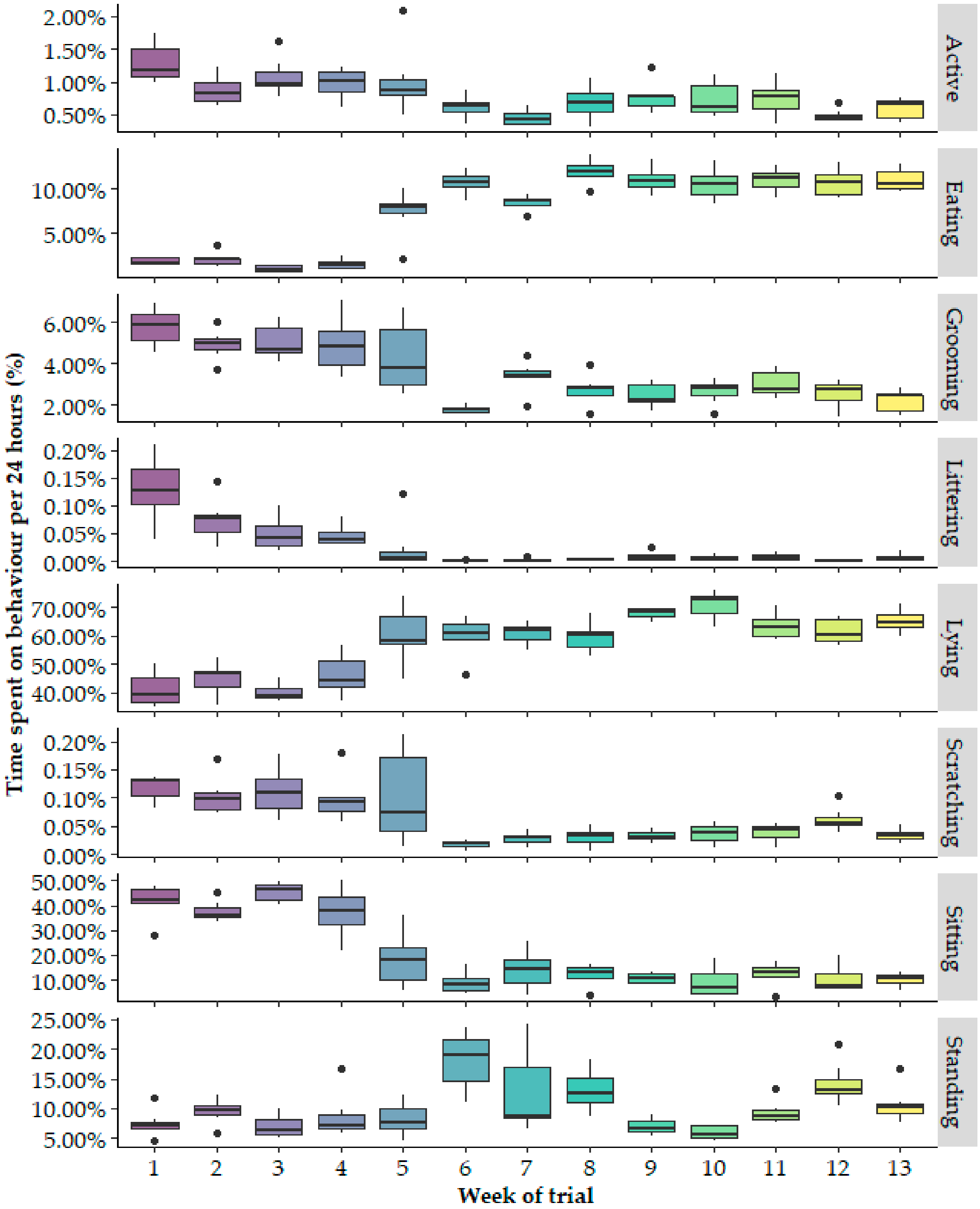

Figure S4. Boxplots of daily proportional behaviour data for Merry.

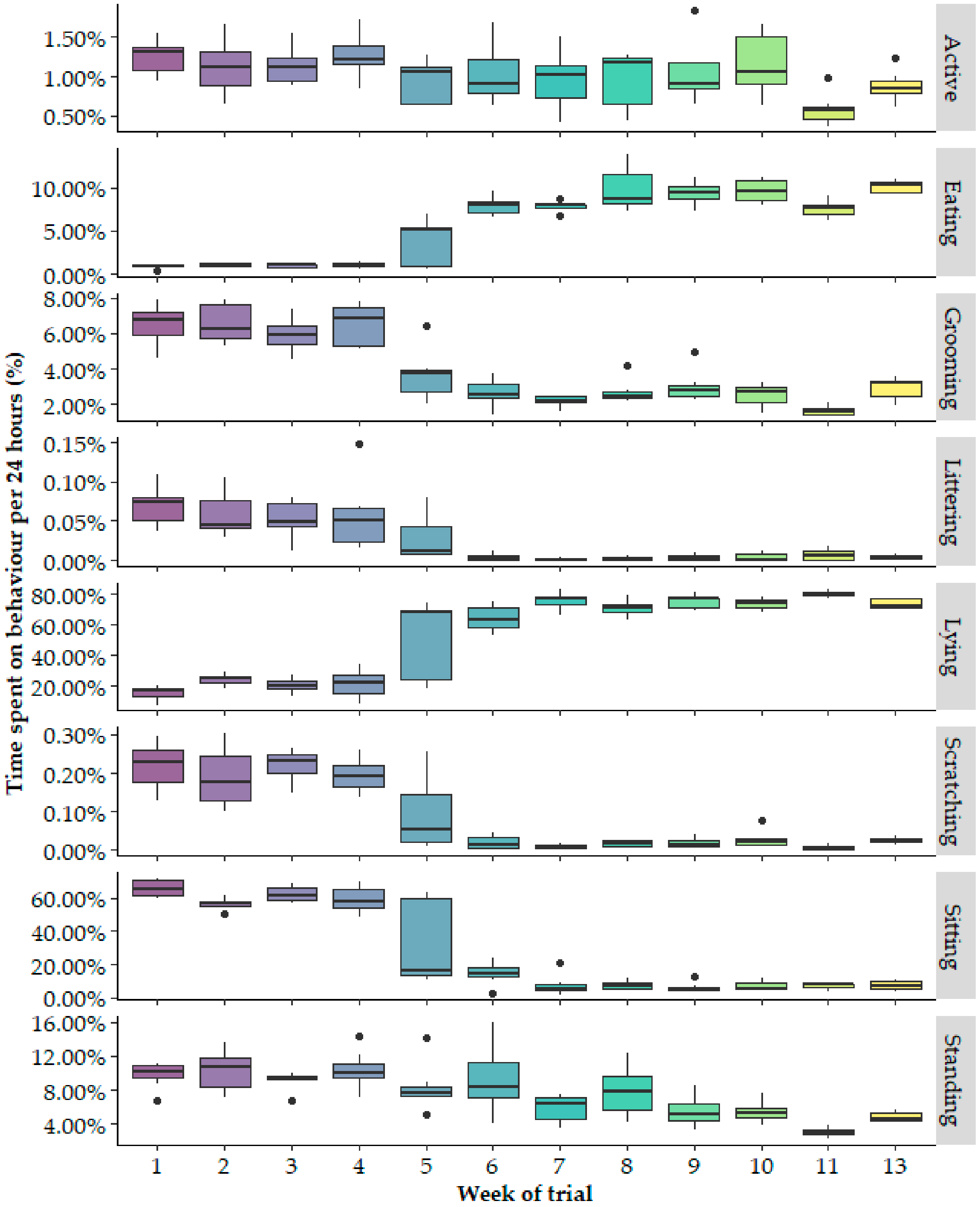

Figure S5. Boxplots of daily proportional behaviour data for Mrs Norris. Data for trial week 12 was removed due to illness.

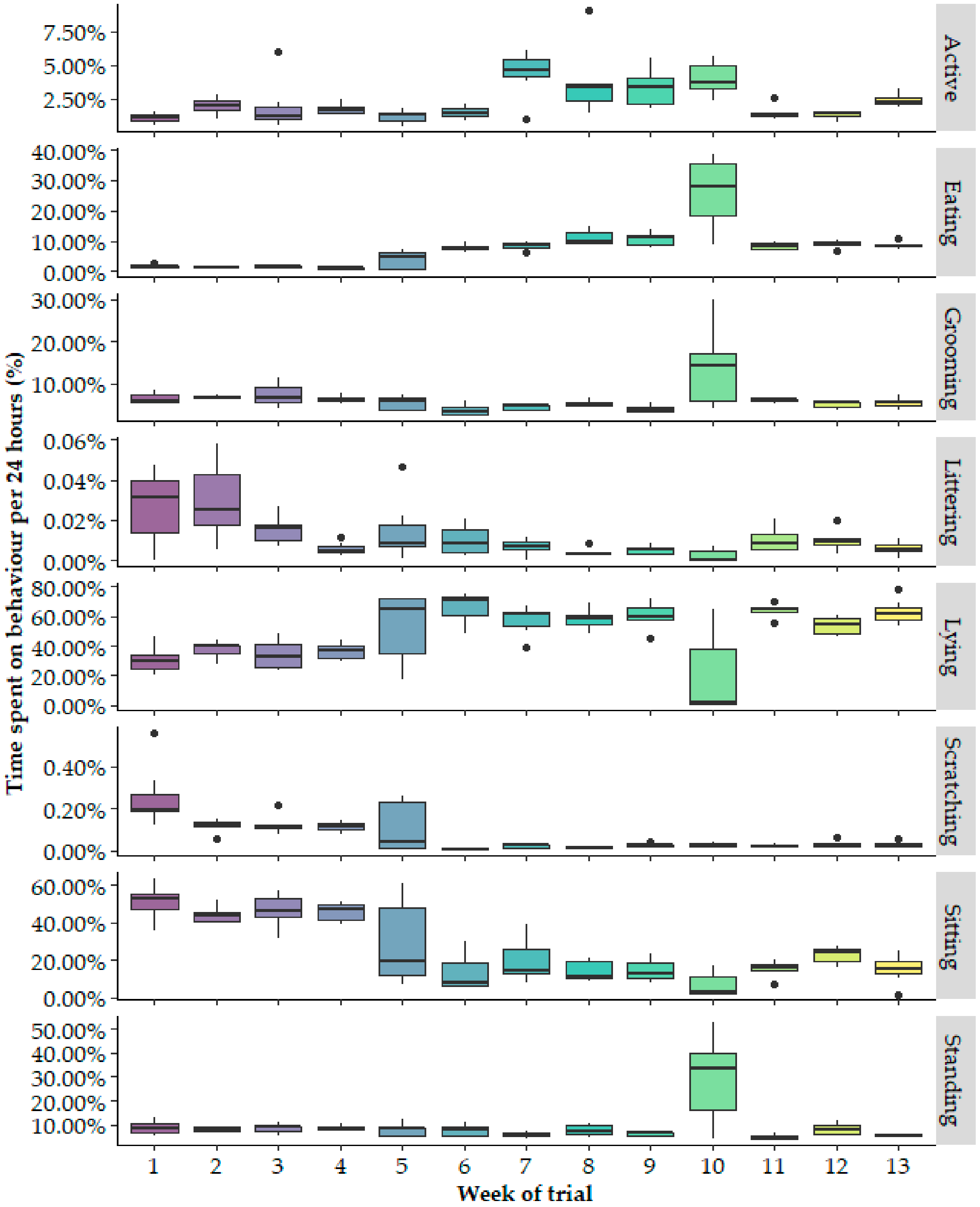

Figure S6. Boxplots of daily proportional behaviour data for Nimbus.

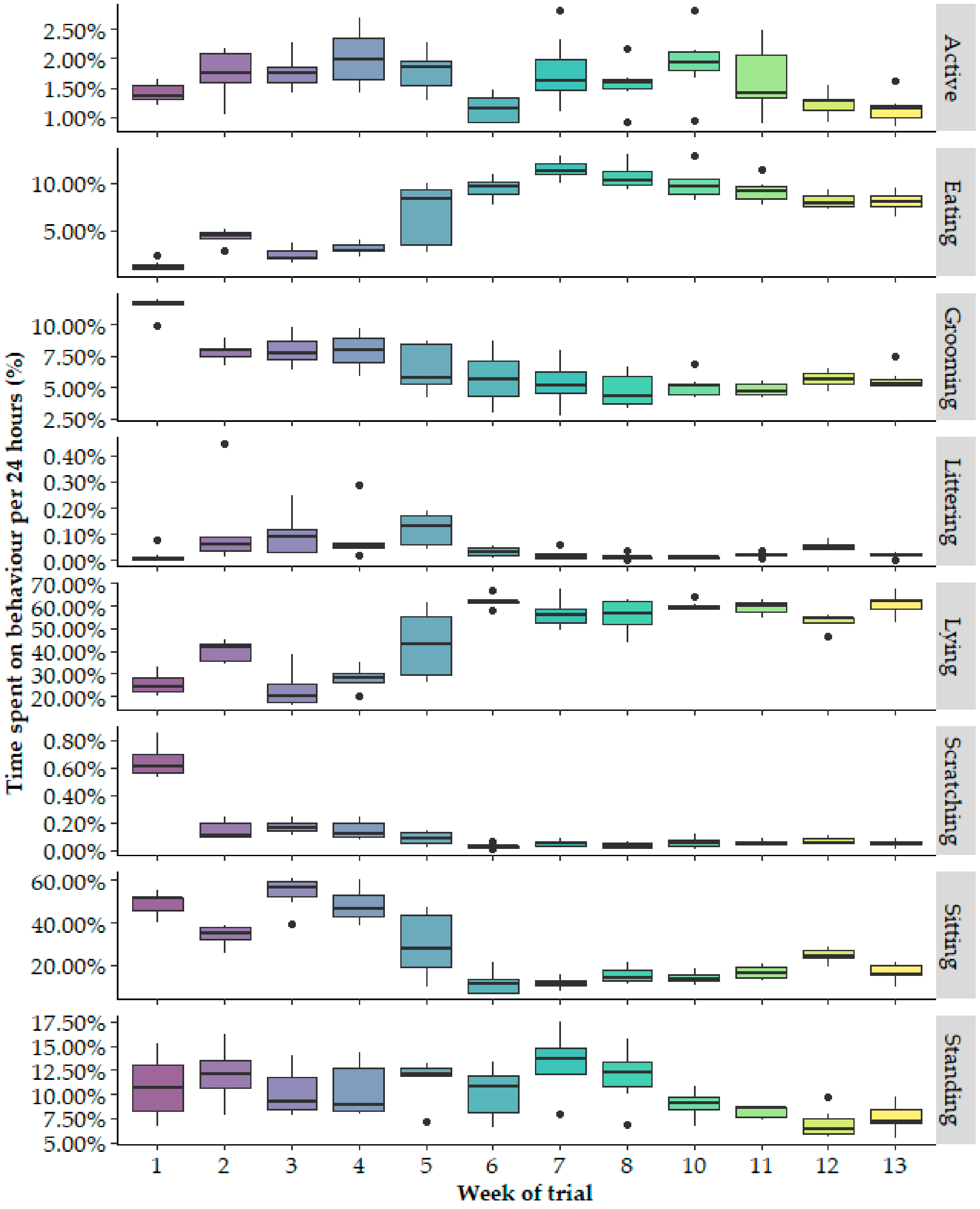

Figure S7. Boxplots of daily proportional behaviour data for Scabbers. Data for trial week 9 was removed due to illness.

Supplementary Material 2

Daylength ranged from 9 hours and 17 minutes in winter to 15 hours and 3 minutes in summer (Figure S8). The lowest temperature recorded was -2.3 °C, and the highest was 34.5 °C. The lowest monthly average temperature recorded was 7.0 °C in August, while the highest was 19.0 °C in January (Figure S8). The temperature gradually decreased from March 2023 until August 2023, after which it gradually increased until January 2024. In autumn, the average temperature was 13.4 °C (range from 1.1 °C to 28.1 °C), in winter 7.9 °C (-2.3 °C to 18.2 °C), in spring 12.2 °C (-0.7 °C to 27.5 °C) and in summer 17.8 °C (3.4 °C to 34.5 °C).

The average relative humidity was lowest in summer (55.8%), followed by spring (60.0%), autumn (65.8%) and winter (67.9%; Figure S8). The maximum recorded relative humidity was 79% across all seasons. The lowest recorded relative humidity for autumn, winter, spring and summer were 36%, 37%, 16% and 18%, respectively.

Spring was the windiest season, with an average wind speed of 1.77 m/s and high of 12.5 m/s. Autumn was the least windy season, with an average of 0.93 m/s and high of 8.5 m/s (Figure S8). The average windspeeds in winter and summer were 0.95 m/s and 1.21 m/s, respectively, with the highest recorded 11.6 m/s and 9.4 m/s, respectively.

The THW-index followed the same pattern as temperature (Figure S8). The averages for the THW-index for autumn, winter, spring and summer were 12.8 °C, 7.2 °C, 11.0 °C and 17.1 °C, respectively.

The wettest season was autumn, with a total of 294 mm of rainfall, while the driest season was summer, with a total of 106 mm (Figure 7). A total of 234 mm and 288 mm of rain fell in winter and spring, respectively.

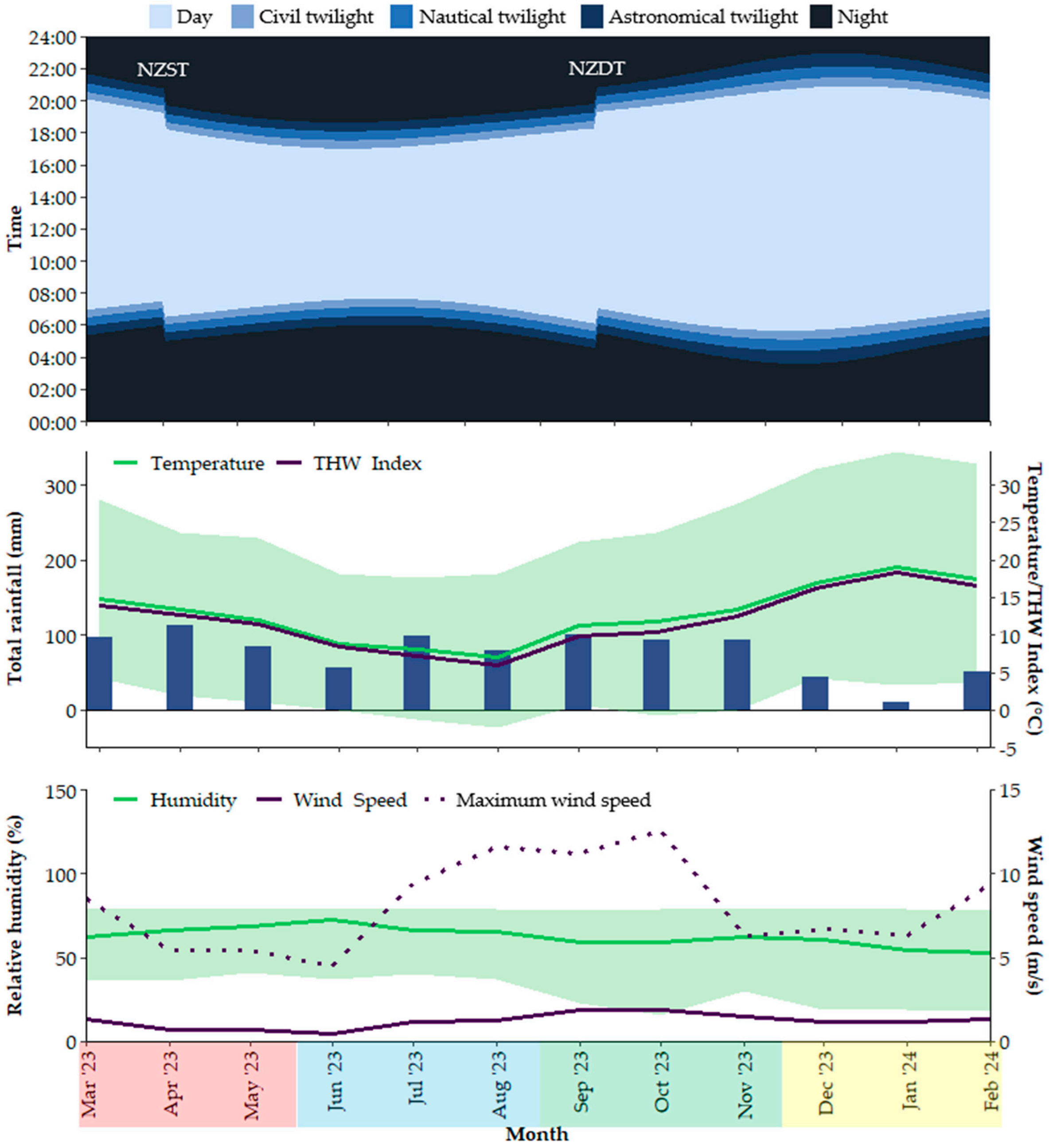

Figure S8. Change in daylength and monthly averages for total rainfall, temperature (± minimum and maximum), THW index, relative humidity (± minimum and maximum) and wind speed (+ maximum) from March 2023 till February 2024. Seasons are indicated along the x-axis with colours (red = autumn, blue = winter, green = spring, yellow = summer). NZST = New Zealand Standard Time, NZDT = New Zealand Daylight Time.
